# Inhalation Administration of the Bicyclic Ethers 1,8- and 1,4-cineole Prevent Anxiety and Depressive-Like Behaviours in Mice

**DOI:** 10.3390/molecules25081884

**Published:** 2020-04-18

**Authors:** Godfried Dougnon, Michiho Ito

**Affiliations:** Department of Pharmacognosy, Graduate School of Pharmaceutical Sciences, Kyoto University, 46-29 Yoshida-Shimoadachi-cho, Sakyo-ku, Kyoto 606-8501, Japan; dougnon.tchetonnougbo.26v@st.kyoto-u.ac.jp

**Keywords:** cineole, anxiety, depression, light-dark box test, marble-burying test, forced swimming test, tail suspension test

## Abstract

The anxiolytic and antidepressant-like activities of the naturally occurring monoterpene 1,8-cineole and its structural isomer 1,4-cineole were evaluated in mice via inhalation administration at doses ranging from 4 × 10^−6^ to 4 × 10^−1^ mg per 400 μL of triethyl citrate. Mice were tested for anxiety-like behaviours by using the light–dark box test (LDB) and marble-burying test (MBT) and for depression-like symptoms by using the forced swimming test (FST) and tail suspension test (TST). Diazepam and fluoxetine were used as standard drugs for anxiolytic and antidepressant tests, respectively. The results showed that 1,8-cineole at 4 × 10^−4^ mg, and 1,4-cineole at 4 × 10^−4^ and 4 × 10^−3^ mg significantly increased the amount of time spent in the light box and the number of entries in the light box in the LDB as well as reduced the number of marbles buried in the MBT relative to those in the control, suggesting an anxiolytic effect. Similarly, 1,8-cineole at 4 × 10^−4^ and 4 × 10^−2^ mg and 1,4-cineole at doses of 4 × 10^−4^ to 4 × 10^−2^ mg significantly reduced immobility times in the FST and TST relative to those of the control, suggesting an antidepressant activity. The role of the GABA_A_/benzodiazepine receptor system in the anxiolytic effects of 1,8- and 1,4-cineole was investigated through co-administration of flumazenil, a GABAergic system antagonist. Flumazenil reversed the effects of diazepam and 1,8-cineole, suggesting that 1,8-cineole affects the GABA_A_/benzodiazepine receptors. Collectively, the results suggest that inhaled 1,8- and 1,4-cineole prevented anxiety and depressive-like symptoms in classic mice models.

## 1. Introduction

Anxiety is one of the most common mental illnesses and affects more than one-eighth of the adult population worldwide [1]. A pharmacotherapeutic approach is typically used to treat anxiety, but these therapies are associated with several side effects, such as memory disturbance, interactions with other drugs, and dependence. Another important psychiatric condition is depression, with a substantial morbidity and high suicide rate. The medicines currently used to treat depression often demonstrate shortcomings, such as slow onset of action, low response rates, and drug resistance development, which limits their utilisation. For many years, anxiety and depression were considered to be separate pathologies, but presently, the use of benzodiazepines for the treatment of anxiety only is being slowly replaced with antidepressants that can treat depression and major anxiety disorders [2]. Aside from their side effects and limited utilisation, these medicines contribute to a high economic burden of disease and are long-term or life-long treatments. Considering these limitations, there is a need for novel psychopharmacological approaches that are efficient with fewer side effects. Recently, natural products have been considered to be promising for the treatment of anxiety and depression, and several studies have investigated essential oils from plant materials because they have demonstrated excellent results in the central nervous system (CNS), such as an anxiolytic, relaxant, sedative, antinociceptive or antidepressant effects [3,4,5].

**Figure 1:**
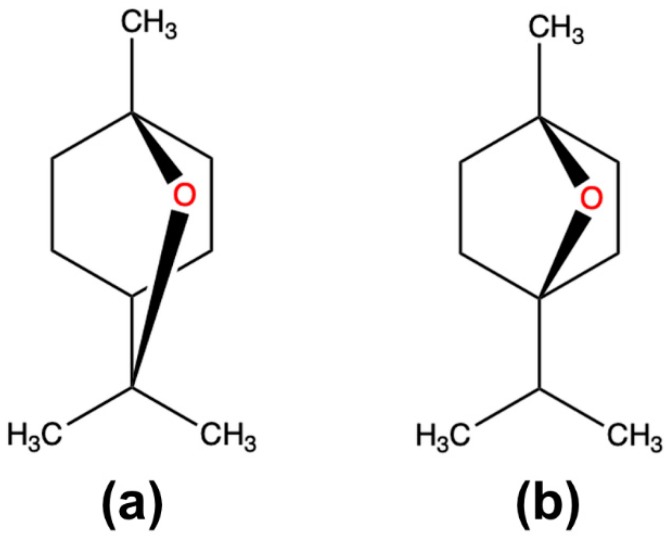
Structural formula of (**a**) 1,8-cineole and (**b**) 1,4-cineole.

Eucalyptol, which is 1,8-cineole or 1,3,3-trimethyl-2-oxabicyclo [2.2.2] octane (Figure 1a), is a monoterpene ether present in essential oils derived from many plants, such as eucalyptus and rosemary. Various pharmacological activities, such as sedative, antihypertensive, anti-inflammatory and antinociceptive effects, of 1,8-cineole have been demonstrated [6,7,8]. A minor component of plant extracts containing 1,8-cineole that is found in cardamom and *Piper cubeba* is 1,4-cineole or 1-methyl-4-propan-2-yl-7-oxabicyclo[2.2.1] heptane (Figure 1b), a natural monoterpene stereoisomer of 1,8-cineole, which has also exhibited interesting bioactivity, such as an anxiolytic-like action in mice via oral administration, and has been shown to be a precursor for microbial hydroxylation [9,10].

Emerging research on the biological effects of 1,8- and 1,4-cineole has shown promising results in humans and animals [11,12]. Studies have shown the anxiolytic or antidepressant-like activity of monoterpenes [3,9,13,14,15,16], but to the best of our knowledge, no studies have been published about the activities of 1,8- and 1,4-cineole in ameliorating anxiety and depressive-like symptoms in mice via inhalation administration.

Therefore, in the present study, we evaluated the anxiolytic-like effects of 1,8- and 1,4-cineole by using two classic mice behavioural models of anxiolytic drug screening known as the light–dark box test (LDB) and marble-burying test (MBT), studied the possible involvement of the GABAergic transmission system in the anxiolytic activities of 1,8- and 1,4-cineole by using flumazenil (FLU; an antagonist of the GABAergic transmission system), evaluated the antidepressant activities of 1,8- and 1,4-cineole by using the forced swimming test (FST) and tail suspension test (TST), which are mice models of depression, and assessed any possible deficit in neuromuscular function that could bias the behavioural activities by performing the horizontal wire test (HWT) in mice.

## 2. Results and Discussion

In a previous study, we demonstrated that 1,8-cineole, one of the major compounds of the essential oil extracted from *Lantana camara* L. leaves, exhibited good sedative activity via inhalation in mice [6]. Because sedative activity is often associated with anxiolytic or antidepressant-like activity [3,14,17,18], further investigations of the effects of 1,8-cineole and its related compounds on the CNS were required. This investigation was based on a structure-activity study showing that 1,8-cineole and its stereoisomer 1,4-cineole prevented anxiety and depressive-like symptoms in mice. To test this hypothesis, classic mice models of anxiety and depression, including the LDB, MBT, FST, TST, open-field test (OFT) and HWT, were used. These tests are based on the exposure of animals to a stressful condition and a specific test for measuring behavioural and physiological responses. In all experiments, the mice were administered inhalation doses ranging from 4 × 10^−6^ to 4 × 10^−1^ mg 1,8- or 1,4-cineole dissolved in 400 μL of triethyl citrate (TEC) individually on the basis of previous experiments [3,14,19].

### 2.1. Anxiolytic-Like Activities of 1,8- and 1,4-cineole by Using the LDB

**Figure 2:**
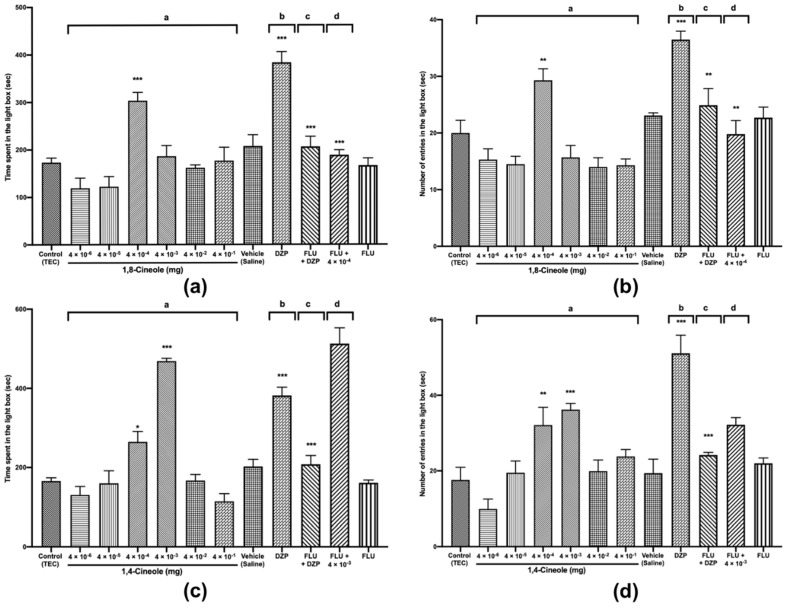
The light–dark box test (LDB) of mice treated with 1,8- (**a**,**b**) or 1,4-cineole (**c**,**d**) (4 × 10^−6^ to 4 × 10^−1^ mg), diazepam (0.5 mg/kg) or flumazenil (2.5 mg/kg). The parameters analysed were the time spent in the light box (TSLB) (**a**,**c**) and the number of entries in the light box (NELB) (**b**,**d**). The values are given as the mean ± SEM of 10 mice. The letter “a” indicates a significant difference when compared with the control (TEC—triethyl citrate); the letter “b” indicates a significant difference when compared with the vehicle (saline); the letter “c” indicates a significant difference when compared with diazepam; the letter “d” indicates a significant difference when compared with 1,8-cineole 4 × 10^−4^ mg or 1,4-cineole 4 × 10^−3^ mg. Statistical differences vs. the control group were calculated by using Student’s t test or one-way analysis of variance (ANOVA) followed by Dunnett’s test. (* *p* < 0.05, ** *p* < 0.01, *** *p* < 0.001).

The LDB is considered to be one of the most widely validated tests for assaying the activities of anxiolytic agents. Reportedly, in the LDB, anxiolytic drugs tend to increase the number of entries in the light box (NELB) and the time spent in the light box (TSLB) [20]. Our results show that diazepam 0.5 mg/kg *i.p.*, used as a positive control, significantly increased TSLB and NELB by 84% and 57%, respectively, relative to the levels in the vehicle (saline) group. A dose of 1,8-cineole 4 × 10^−4^ mg significantly increased TSLB by 75% relative to the level in the control group (*F* (11,108) = 14.79, *p* < 0.001) (Figure 2a); however, it was decreased by 20% relative to that after dosing with diazepam. Similarly, 4 × 10^−4^ mg 1,8-cineole significantly increased NELB by 40% relative to the level in the control group (*F* (11,108) = 13.37, *p* < 0.001) (Figure 2b), but decreased it by 17% relative to the level when using diazepam. These results validated the experiment and suggested an anxiolytic-like activity of 1,8-cineole slightly inferior to that of diazepam.

Our findings are supported by the study of Kim et al. [21], who showed that inhaled 1,8-cineole, significantly reduced preoperative anxiety in humans. Similarly, administration of the essential oil from *Achillea wilhelmsii* C. Koch, which contains 1,8-cineole as a major compound (20.8%), showed anxiolytic activity [4].

We found that 1,4-cineole at doses of 4 × 10^−4^ and 4 × 10^−3^ mg significantly increased TSLB by 60% and 182%, respectively (*F* (11,108) = 38.20, *p* < 0.001) (Figure 2c), and significantly increased NELB by 83% and 110%, respectively, relative to the levels in the control (*F* (11,108) = 13.11, *p* < 0.001) (Figure 2d). Additionally, the effect of 1,4-cineole 4 × 10^−3^ mg was better than that of 4 × 10^−4^ mg in both TSLB and NELB and 23% greater than that of diazepam. These results indicate a potential anxiolytic effect of 1,4-cineole. Our inhalation administration of 1,4-cineole showed results similar to those of Gomes et al. [9], who demonstrated that the oral administration of 1,4-cineole 400 mg/kg had anxiolytic activity in mice. These results suggested that 1,8- and 1,4-cineole could be used as anxiolytic agents depending on the dose and route of administration.

### 2.2. The Role of the GABA_A_/Benzodiazepine Receptor System in the Anxiolytic Activity of 1,8- and 1,4-cineole

Several mechanisms have been proposed to explain anxiety and depression symptoms, and studies have frequently reported mediation via the GABAergic transmission system [5,22,23]. GABA is the primary inhibitory neurotransmitter in the CNS, and it has been reported that one third of all CNS neurons are thought to be mediated via the GABAergic transmission system [23]. Therefore, we investigated the implication of the GABAergic system transmission in the anxiolytic activity of 1,8- and 1,4-cineole. FLU 2.5 mg/kg *i.p.*, an antagonist of benzodiazepine drugs, was administered together with 1,8-cineole 4 × 10^−4^ mg and 1,4-cineole 4 × 10^−3^ mg in the LDB. During the tests with 1,8-cineole, FLU alone did not alter TSLB and NELB (Figure 2a,b, respectively). However, the pre-treatment of FLU with diazepam significantly reduced TSLB by 45% (*F* (11,108) = 16.79, *p* < 0.001) (Figure 2a) and NELB by 34% (*F* (11,108) = 13.37, *p* < 0.001) (Figure 2b) relative to the tests with diazepam alone. These results confirmed the antagonistic effect of FLU on diazepam. Similarly, the treatment of 1,8-cineole 4 × 10^−4^ mg with FLU significantly reduced TSLB and NELB by 38% and 34%, respectively, relative to the tests with 1,8-cineole 4 × 10^−4^ mg (Figure 2a,b), suggesting an antagonistic effect of FLU on 1,8-cineole. Some authors have previously suggested that 1,8-cineole may exert its activity through the GABAergic transmission system [15,24].

During the tests with 1,4-cineole, the FLU, diazepam, and diazepam + FLU groups showed similar results to those of the tests with 1,8-cineole, which validated the experiments. However, in both TSLB and NELB, the anxiolytic effect of 1,4-cineole 4 × 10^−3^ mg was not reversed by co-administration with FLU (Figure 2c,d), indicating that the anxiolytic effect of 1,4-cineole was not mediated via GABA_A_/benzodiazepine receptors. These results are consistent with those of Gomes et al. [9], who found that 1,4-cineole administered orally to mice did not involve benzodiazepine receptors. Further investigations on 1,4-cineole need to be conducted in future experiments to elucidate the possible role of 5-HT_1A_, D1 or noradrenergic receptors in the anxiolytic activity.

### 2.3. Anxiolytic-Like Activities of 1,8- and 1,4-cineole by Using the MBT

**Figure 3:**
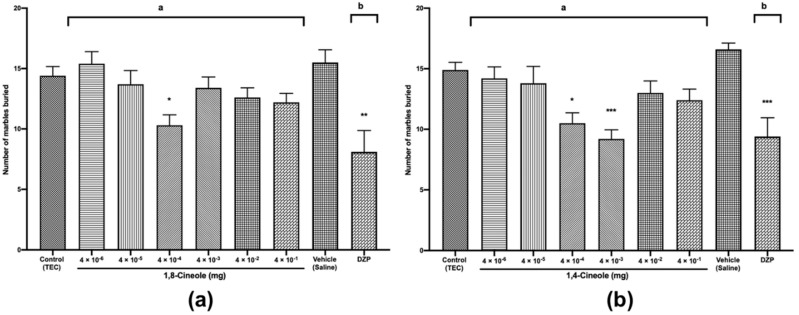
The marble-burying test (MBT) of mice treated with 1,8- (**a**) or 1,4-cineole (**b**) (4 × 10^−6^ to 4 × 10^−1^ mg) or diazepam (0.5 mg/kg). The parameter analysed was the number of marbles buried (NMB). The values are given as the mean ± SEM of 10 mice. The letter “a” indicates a significant difference when compared with the control (TEC); the letter “b” indicates a significant difference when compared with the vehicle (saline). Statistical differences vs. the control group were calculated by using Student’s t test or ANOVA followed by Dunnett’s test. (* *p* < 0.05, ** *p* < 0.01, *** *p* < 0.001).

To further corroborate the anxiolytic activity observed in the LDB, we also performed the MBT in which rodents tend to bury glass marbles. This behaviour is interpreted as related to anxiety; consequently, anxiolytic drugs tend to reduce the number of marbles buried (NMB) [13]. Results similar to those of the LDB were observed for both 1,8- and 1,4-cineole in the MBT. When compared with the NMB of the control, that of the mice treated with 1,8-cineole 4 × 10^−4^ mg was reduced by 26% (*F* (8,81) = 5.297, *p* < 0.001), which indicated an anxiolytic-like effect of 1,8-cineole (Figure 3a). The NMBs of the mice treated with 1,4-cineole at doses of 4 × 10^−4^ and 4 × 10^−3^ mg were reduced by 29% and 38%, respectively (*F* (8,81) = 6.431, *p* < 0.001), relative to those of the control (Figure 3b), indicating an anxiolytic-like activity of 1,4-cineole.

Moreover, 1,4-cineole 4 × 10^−3^ mg had a superior anxiolytic-like effect to that of 1,4-cineole 4 × 10^−4^ mg and was greater than that of diazepam, as observed in the LDB. Similar to 1,8- and 1,4-cineole, other monoterpenes and derivatives of monoterpenes have been demonstrated to possess anxiolytic-like activity, such as that shown by epoxy–limonene administered at 25, 50 and 75 mg/kg *i.p.* in Swiss mice [13] and of carvacryl acetate administered at 50 and 75 mg/kg *i.p.* in mice [16], which demonstrated good anxiolytic activities relative to those of the vehicle.

### 2.4. Sedative Effects of 1,8- and 1,4-cineole by Using the OFT

**Figure 4:**
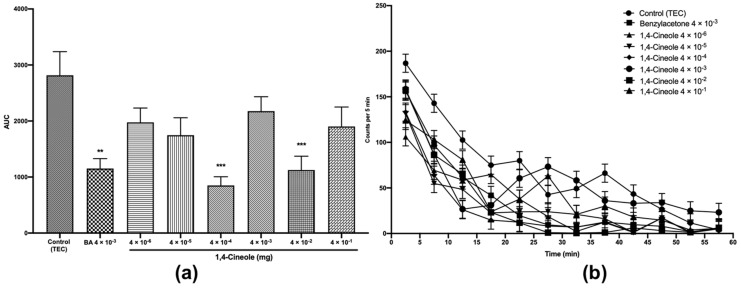
Total spontaneous motor activity (**a**) and locomotor activity transition (**b**) of mice treated with 1,4-cineole (4 × 10^−6^ to 4 × 10^−1^ mg). The values are given as the mean ± SEM of six mice. Statistical differences vs. the control group were calculated by using Student’s t test or ANOVA followed by Dunnett’s test. (** *p* < 0.01, *** *p* < 0.001).

To further investigate the effects of 1,8- and 1,4-cineole on the CNS, we explored the sedative activity of 1,4-cineole in the OFT. The OFT utilises behavioural changes in rodents exposed to a novel environment and has been used to detect sedative activity in mice [3,14,19,25,26,27]. Mice administered 1,4-cineole at doses ranging from 4 × 10^−6^ to 4 × 10^−1^ mg per 400 μL of TEC showed a decrease in locomotor activity at all doses. As shown in Figure 4a, there was a significant decrease in mice locomotor activity at doses of 1,4-cineole 4 × 10^−4^ and 4 × 10^−2^ mg by 70% and 59%, respectively (*F* (7,40) = 5.236, *p* < 0.001), relative to those of the control. Moreover, the analysis of locomotor activity transition (Figure 4b) showed that the sedative effects produced by the doses of 4 × 10^−4^ mg and 4 × 10^−2^ mg 1,4-cineole were the most effective and locomotor activity dropped nearly to zero 15–20 min after inhalation administration.

Controversially, Gomes et al. [9] showed that 1,4-cineole 100 and 200 mg/kg *p.o* did not induce any change in mice locomotor activity. Differences in the results could be due to the differences in the doses administered and routes of administration. Additionally, Gomes et al. [9] performed the OFT for 5 min, whereas we performed it for 60 min. It has been reported that the sedative activity is observed generally 20–30 min after drug administration [3,14,19,25,26,27]. Moreover, Gomes et al. [9] concluded that a higher dose of 1,4-cineole could demonstrate sedative activity because their pentobarbital test indicated a possible sedative effect for 1,4-cineole. The sedative effect of 1,8-cineole was studied in our previous study [6] and it showed a significant decrease in locomotor activity at 4 × 10^−4^ mg. Taken together, our results observed in the LDB, MBT and OFT suggest that 1,8- and 1,4-cineole exhibit anxiolytic effects associated with sedative action in mice. Similar to the activity in 1,8- and 1,4-cineole, anxiolytic activity has been often associated with sedative effects in other terpenoids, such as linalool, nerol, limonene epoxide, and thymol and in essential oils from *Ocimum gratissimum* L., *Telfairia occidentalis*, *Piper guineense,* and *Citrus aurantium* L. [3,13,14,17,18,28,29].

### 2.5. Evaluation of a Possible Peripheral Neuromuscular Blockage by Using the HWT

**Figure 5:**
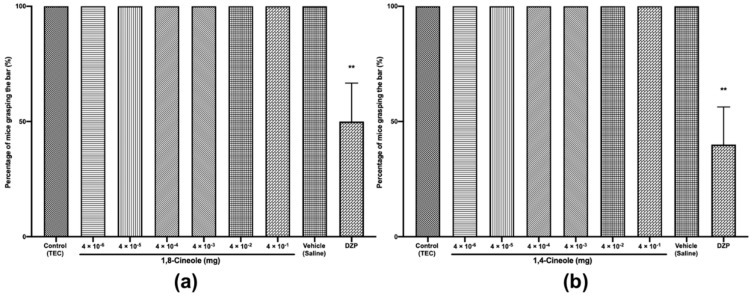
Horizontal wire test (HWT) of mice treated with 1,8- (**a**) or 1,4-cineole (**b**) (4 × 10^−6^ to 4 × 10^−1^ mg) or diazepam (5 mg/kg). The values are given as the mean ± SEM of 10 mice. Statistical differences vs. the control group were calculated by using Student’s t test or ANOVA followed by Dunnett’s test. (** *p* < 0.01).

A deficit in motor coordination would likely affect the performance of the mice in the OFT. Therefore, we investigated the effects of 1,8- and 1,4-cineole in the HWT, a classic animal model used to evaluate peripheral neuromuscular blockage. Our findings show that diazepam 5 mg/kg *i.p.* as a positive control significantly decreased the percentage of mice grasping the wire by 60% and 50% in the tests with 1,8- and 1,4-cineole, respectively, relative to the levels in the control (Figure 5a,b), indicating a myorelaxant effect of diazepam.

In contrast, no change was observed after treatment with 1,8- or 1,4-cineole at doses ranging from 4 × 10^−6^ to 4 × 10^−1^ mg per 400 μL of TEC relative to the levels in the control, indicating a lack of myorelaxation effect at these doses. Consequently, the observed decrease in locomotor activity is probably not related to peripheral neuromuscular blockage but may involve neurons that control CNS activity. Similar results were obtained for the methyl and isopropyl *N*-methylanthranilates from *Choisya ternata* and for quercetin [23,30]. The LD_50_ value for oral administration of 1,8-cineole in mice is 3849 mg/kg [31], whereas the LD_50_ value for 1,4-cineole is 3100 mg/kg in rats [32]. The maximum administered dose in our study was 4 × 10^−1^ mg, which is much lower than the toxic doses. Moreover, abnormalities, such as an increase in urination or defecation, twisting, tremors, seizures, catalepsy and stereotypical behaviours, were not noticed, suggesting that the effect is probably not toxic.

### 2.6. Antidepressant-Like Activities of 1,8- and 1,4-cineole by Using the FST and TST

**Figure 6:**
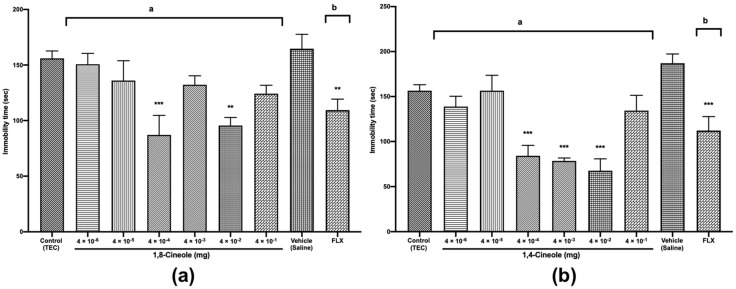
The Forced swimming test (FST) of mice treated with 1,8- (**a**) or 1,4-cineole (**b**) (4 × 10^−6^ to 4 × 10^−1^ mg) or fluoxetine (20 mg/kg). The parameter analysed was immobility time in each test. The values are given as the mean ± SEM of 10 mice. The letter “a” indicates a significant difference when compared with the control (TEC); the letter “b” indicates a significant difference when compared with the vehicle (saline). Statistical differences vs. the control group were calculated by using Student’s t test or ANOVA followed by Dunnett’s test. (** *p* < 0.01, *** *p* < 0.001).

In addition to the anxiolytic-like activities, we investigated 1,8- and 1,4-cineole, for their activity in the most widely used animal models for antidepressant drug screening, the FST [33] and TST [34]. In these tests, mice are forced to swim or are hung by their tails in an inescapable situation; the mice first demonstrate vigorous activity trying to escape the threatening environment and finally become immobile as a symptom of behavioural despair. Substances that decrease immobility time are known to demonstrate antidepressant properties in humans. In the FST, 1,8-cineole at doses of 4 × 10^−4^ and 4 × 10^−2^ mg induced a significant decrease in the immobility time of mice by 44% and 39%, respectively, relative to the times in the control group (*F* (8,81) = 5.400, *p* < 0.001) (Figure 6a).

Treatment with the antidepressant drug fluoxetine (FLX) at 20 mg/kg *p.o.*, used as a positive control, also significantly reduced the immobility time by 40% relative to the time in the vehicle (saline) group. Additionally, 1,8-cineole at doses of 4 × 10^−4^ and 4 × 10^−2^ mg had greater antidepressant effects by 21% and 13%, respectively, relative to those for FLX. Antidepressant effects were previously observed in mice after the oral administration of *R. officinalis* containing 45% 1,8-cineole [35]. On the other hand, no antidepressant activity was detected for 1,8-cineole 1 mg/kg administered intraperitoneally [15]; however, the authors suggested that the single-dose regimen of 1,8-cineole 1 mg/kg *i.p.* could be insufficient to potentially produce an antidepressant effect. As shown in Figure 6b, 1,4-cineole at doses of 4 × 10^−4^ to 4 × 10^−2^ mg induced dose-dependent significant decreases in the immobility times of mice by 46%, 49% and 56%, respectively, relative to those in the control group (*F* (8,81) = 10.60, *p* < 0.001). Compared with the effect of FLX, the effects of 1,4-cineole at doses of 4 × 10^−4^ to 4 × 10^−2^ mg were 24%, 29% and 39% greater, indicating the potent antidepressant activity of 1,4-cineole. In contrast to our study, Gomes et al. [9] found possible depressive activity of 1,4-cineole 400 mg/kg *p.o*. The differences in the results could also be explained by differences in the administered doses and routes of administration. Sousa et al. [36] made the same observations, suggesting that the administration route or difference in experimental models could explain differences in the pharmacological effects.

**Figure 7:**
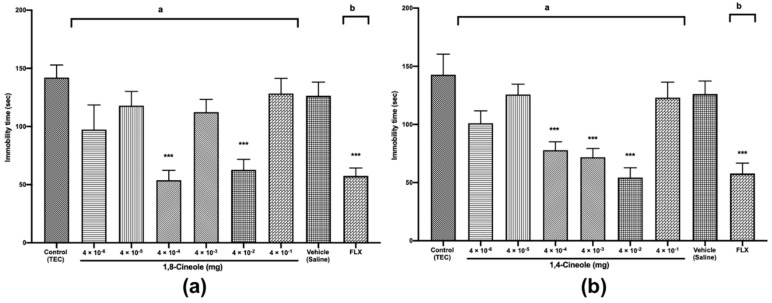
Tail suspension test (TST) of mice treated with 1,8- (**a**) or 1,4-cineole (**b**) (4 × 10^−6^ to 4 × 10^−1^ mg) or fluoxetine (20 mg/kg). The parameter analysed was immobility time in each test. The values are given as the mean ± SEM of 10 mice. The letter “a” indicates a significant difference when compared with the control (TEC); the letter “b” indicates a significant difference when compared with the vehicle (saline). Statistical differences vs. the control group were calculated by using Student’s t test or ANOVA followed by Dunnett’s test. (*** *p* < 0.001).

In the TST, as shown in Figure 7a, similar to the results observed in the FST, 1,8-cineole at doses of 4 × 10^−4^ and 4 × 10^−2^ mg significantly reduced the immobility times in mice by 62% and 55%, respectively, relative to those in the control group (*F* (8,81) = 7.563, *p* < 0.001).

Figure 7b shows that 1,4-cineole at doses of 4 × 10^−4^ to 4 × 10^−2^ mg significantly decreased the immobility times in mice by 45%, 49% and 62%, respectively, relative to those of the control group (*F* (8,81) = 9.290, *p* < 0.001). The TST for 1,8- and 1,4-cineoles showed results similar to those of the FST, confirming the validity of our experiments. Similar to the activities of 1,8- and 1,4-cineole, antidepressant activity was previously demonstrated for terpinen-4-ol contained in *Origanum majorana* [37], beta-pinene and linalool (principal constituents of *Litsea glaucescens* [38]), and for the essential oil of *Rosmarinus officinalis* [35].

### 2.7. Relationship Structure-Activity of 1,8- and 1,4-cineole

**Figure 8:**
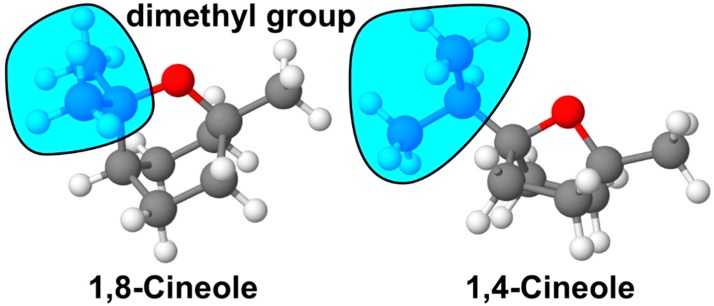
Tri-dimensional conformation showing the position of the free dimethyl group (circled in blue) of 1,8-cineole compared with that of 1,4-cineole.

The two cineole isomers investigated in the present study have identical molecular weights (MW = 154.25 g/mol) and molecular formulas (C_10_H_18_O) but they differ in the position of the oxygen bridge connecting atoms 1 and 4 in 1,4-cineole or atoms 1 and 8 in 1,8-cineole. However, despite the structural similarity, their biological actions and mechanisms were different, which makes it interesting to compare the possible relationships between their physical and biochemical characteristics and their activities. The position of a functional group in a compound is reported to likely have activation or inhibitory effects on their activity. In this way, the study of Miyoshi et al. [25] on benzylacetone and derivatives demonstrated that a series of benzylacetone isomers differing only in the position of the ketone group showed different sedative activity levels, with the most sedative isomers being compounds with a ketone group on the carbon adjacent to C3. Additionally, aromatic compounds with isomeric structures have previously demonstrated various effects via the activation of the olfactory bulb [39]. The boiling point of 1,8-cineole is 176–177 °C, which is higher than 172–174 °C for 1,4-cineole. Compounds with higher boiling points often have reduced vaporisation that may contribute to weakening of behavioural activity, which could explain the greater activity of 1,4-cineole than that of 1,8-cineole. Differences in conformation can also affect the activity of compounds. Miyoshi et al. [25] demonstrated that different steric configurations of the oxygen atom affected the activity of 4-phenyl-2-butanol. Another study also demonstrated that *cis*-isomers were 10-fold more potent than *trans*-isomers of compounds [26]. Regarding 1,8- and 1,4-cineole, the spatial orientation of the dimethyl side chain differed between the two molecules, as shown in Figure 8.

In 1,4-cineole, the dimethyl group is free and located far from the hexyl ring, whereas in 1,8-cineole, the dimethyl group is involved in the formation of a heterocyclic ring [40]. This difference made 1,4-cineole a more flexible structure with a freely rotating dimethyl group that can interact with several types of olfactory receptors. Therefore, the differences in structural, biochemical and physical properties could explain the differences in the behavioural activities of 1,8- and 1,4-cineole.

## 3. Materials and Methods

### 3.1. Chemical and Reagents

Triethyl citrate (purity > 98%, Merck, Kenilworth, NJ, USA), a non-sedating odourless solvent, was used to dissolve the fragrant components. Benzylacetone (purity > 95%, Tokyo Chemical Industries, Ltd., Tokyo, Japan), flumazenil (purity > 98%, Wako Pure Chemical Industries, Ltd., Osaka, Japan), diazepam (purity > 98%, Wako Pure Chemical Industries, Ltd., Osaka, Japan) and fluoxetine (purity > 98%, Tokyo Chemical Industries, Ltd., Tokyo, Japan) were used as positive controls. 1,4-Cineole (purity > 85%) was obtained from Acros Organics (New Jersey, NJ, US). 1,8-Cineole (purity > 85%) was purchased from Wako Pure Chemical Industries, Ltd., Osaka, Japan. All chemicals used were of the highest grade available.

### 3.2. Experimental Animals

Four-week-old male ddY mice (20–30 g) were purchased from Japan SLC (Shizuoka, Japan). The animals were housed in colony cages under a 12 h/12 h light/dark cycle at 25 ± 2 °C and a relative humidity of 50–60%. They were fed pellet chow and water ad libitum and allowed to accommodate to these conditions for 1 week before the experiments. Animal experiments were conducted following the recommendations of the Animal Research Committee of Kyoto University, Kyoto, Japan (approval number, 2014-14-3; first approved 27 December 2014 and renewed annually until 14 March 2018). Experimental procedures involving animals and their care were conducted in accordance with the institutional guidelines and in compliance with the Fundamental Guidelines for the Proper Conduct of Animal Experiments and Related Activities in Academic Research Institutions under the jurisdiction of the Ministry of Education, Culture, Sports, Science and Technology, Japan (2006). All experiments were conducted between 10:00 and 17:00 under identical conditions.

### 3.3. Behavioural Experiments

Anxiety symptoms were evaluated by using the LDB and MBT, while depressive-like behaviours were investigated by using the FST and TST, as previously demonstrated [3,20,33,34] with minor modifications. Inhalation administration was performed as described in the OFT. The sedative effects and motor coordination were evaluated by using the OFT and HWT, respectively [3,30]. To avoid any potential bias in the experiments, each treatment group was recorded by using a video camera and scored by a trained observer blind to the treatment. In all our experiments, each animal was used only once.

#### 3.3.1. Light–Dark Box Test (LDB)

The LDB is a widely used behavioural test for evaluating the anxiolytic effects of drugs. The test is based on the innate aversion of rodents to brightly lit areas and on their spontaneous exploratory behaviour in response to a novel environment and to light [20]. The apparatus consisted of two equally sized compartments (30 × 30 × 34 cm each): a light area illuminated by a 60-watt desk LED lamp, and a dark area blackened with black plastic sheets. The two compartments were separated by a black wall with an aperture (small doorway) in its centre (5 × 5 cm) to allow passage from one compartment to the other. In both compartments 1,8- or 1,4-cineole was charged for 60 min in accordance with the open-field test. Thereafter, mice (*n* = 10/group) were individually placed in the centre of the lit area facing the tunnel, and activity was recorded by using a video camera during a 15-min test period, after which the number of entries in the light box (NELB) and time spent in the light box (TSLB) were counted. Diazepam 0.5 mg/kg *i.p.* was used as a positive control, as previously described [3].

#### 3.3.2. Marble-Burying Test (MBT)

The MBT is an effective method for testing the anxiolytic-like properties of a particular substance. The test is based on the defensive burying behaviour observed in rodents in response to aversive stimuli, such as shock; noxious food; or unanimated objects, such as glass marbles [41]. Mice (*n* = 10/group) were individually introduced into the apparatus that consisted of a Plexiglas cage (42 × 34 × 15 cm) with a floor filled with a 5-cm deep layer of sawdust and containing 20 distributed glass marbles (15-mm diameter). Each test lasted 30 min, and a marble was considered as hidden when it was at least two-thirds covered by sawdust. The sawdust and marbles were washed with soap and water, cleaned with ethanol 70% and dried with a paper towel, and then the next mouse was tested.

#### 3.3.3. Open-Field Test (OFT)

The sedative effects of 1,4-cineole on mice were evaluated using an OFT, as previously described [3,14,19,25,26,27]. Administered doses were expressed as milligrams of sample per 400 μL of TEC following previous experiments [14,25,42]. Four pieces of filter paper were placed in the four corners of the inner walls of the glass cage (60 cm wide, 30 cm long, 34 cm high) by using adhesive tape. On each piece of filter paper, 1,4-cineole was deposited, and the cage was closed so that the vapour pervaded by natural diffusion. Sixty minutes after charging the sample, the mice (*n* = 6/group) were individually placed in the centre of the cage and subjected to video surveillance for another 60 min. During monitoring, the number of times a mouse crossed lines drawn at 10-cm intervals on the floor of the cage was counted every 5 min. The area under the curve (AUC) of locomotor activity counts per 5 min (*Y*-axis) and time (*X*-axis), representing total locomotor activity, was calculated according to the trapezoidal rule.

#### 3.3.4. Horizontal Wire Test (HWT)

The HWT estimates motor coordination and muscle relaxation [30]. The test was performed by treating the mice according to a slight modification of the method described by Bonetti et al. [43]. The mice were lifted by their tails and allowed to grasp a horizontally strung wire (2-mm diameter, 30-cm long) placed 25 cm above a table with their forepaws, after which they were released. The number of mice from each treatment group (*n* = 10/group) that did not grasp the wire with their forepaws or actively grasp the wire with at least one hind paw within a 10-sec period was recorded. Diazepam (5 mg/kg, *i.p.*) was used as a positive control and administered 30 min before the test.

#### 3.3.5. Forced Swimming Test (FST)

The FST is the most widely used test for the assessment of antidepressant activity. The procedure used is the same as previously described by Porsolt et al. [44] with some minor modifications. After treatment with saline, fluoxetine (20 mg/kg, *p.o.*), TEC, or 1,8- or 1,4-cineole, the mice (*n* = 10/group) were individually forced to swim in a transparent Plexiglas cylinder (40-cm high and 20-cm in diameter) containing a 15-cm depth of water at 25 ± 2 °C. The water was renewed after each mouse was tested. During the session, immobility time was recorded by using a video camera. The total duration of immobility was measured during the last 4 min of a single 6-min test session. The mice were considered to be immobile when they remained floating motionless or when they only made movements necessary to keep their heads above water.

#### 3.3.6. Tail Suspension Test (TST)

The TST is a widely used behavioural model for testing the effect of antidepressant agents. The test was carried out by using a method described by Steru et al. [34]. Mice (*n* = 10/group) were individually suspended from the edge of a suspension box (63-cm high) by an adhesive tape placed approximately 1 cm from the tip of the tail. The mice were considered to be immobile when they stopped making any struggling movements and hung passively. The immobility time was recorded for a period of 6 min. Fluoxetine (20 mg/kg *p.o.*) was administered as positive control 1 h prior to testing, to confirm the validity of the apparatus.

### 3.4. Statistical Analysis

All values are expressed as the mean ± standard error of mean (SEM). Statistical analyses were performed by using Student’s t-test or one-way analysis of variance (ANOVA) followed by Dunnett’s multiple comparison test. GraphPad InStat software version 7 (GraphPad Software, Inc.) was used to perform all statistical analyses. *p* values < 0.05 were considered to be indicative of statistical significance.

## 4. Conclusions

Inhalation administration is a non-harmful method of administration that can be applied to any patient without distinction of age or mental or physical condition. This study demonstrated that 1,8- and 1,4-cineole possessed good sedative, anxiolytic, and antidepressant activities when administered to mice via inhalation. The study findings suggest that 1,8- and 1,4-cineole could be considered for the treatment of CNS-related pathologies, such as post-traumatic stress disorder, attention deficit hyperactivity disorder, insomnia, anxiety or depression.
